# Feasibility Study of a Small Animal PET Insert Based on a Single LYSO Monolithic Tube

**DOI:** 10.3389/fmed.2018.00328

**Published:** 2018-11-28

**Authors:** Antonio J. Gonzalez, Stuart S. Berr, Gabriel Cañizares, Andrea Gonzalez-Montoro, Abel Orero, Carlos Correcher, Ahmadreza Rezaei, Johan Nuyts, Filomeno Sanchez, Stan Majewski, Jose M. Benlloch

**Affiliations:** ^1^Detector for Molecular Imaging Lab (DMIL), Instituto de Instrumentacion para Imagen Molecular (i3M), Centro Mixto CSIC - Universitat Politècnica de València, Valencia, Spain; ^2^Department of Radiology and Medical Imaging, University of Virginia, Charlottesville, VA, United States; ^3^Bruker NMI, Valencia, Spain; ^4^Nuclear Medicine and Medical Imaging Research Center KU Leuven, Leuven, Belgium; ^5^MoSAIC, Molecular Small Animal Imaging Center, KU Leuven – University of Leuven Leuven, Belgium

**Keywords:** Positron emission tomography, hybrid PET-MR, monolithic crystal, SiPM, Preclinical imaging

## Abstract

There are drawbacks with using a Positron Emission Tomography (PET) scanner design employing the traditional arrangement of multiple detectors in an array format. Typically PET systems are constructed with many regular gaps between the detector modules in a ring or box configuration, with additional axial gaps between the rings. Although this has been significantly reduced with the use of the compact high granularity SiPM photodetector technology, such a scanner design leads to a decrease in the number of annihilation photons that are detected causing lower scanner sensitivity. Moreover, the ability to precisely determine the line of response (LOR) along which the positron annihilated is diminished closer to the detector edges because the spatial resolution there is degraded due to edge effects. This happens for both monolithic based designs, caused by the truncation of the scintillation light distribution, but also for detector blocks that use crystal arrays with a number of elements that are larger than the number of photosensors and, therefore, make use of the light sharing principle. In this report we present a design for a small-animal PET scanner based on a single monolithic annulus-like scintillator that can be used as a PET insert in high-field Magnetic Resonance systems. We provide real data showing the performance improvement when edge-less modules are used. We also describe the specific proposed design for a rodent scanner that employs facetted outside faces in a single LYSO tube. In a further step, in order to support and prove the proposed edgeless geometry, simulations of that scanner have been performed and lately reconstructed showing the advantages of the design.

## Introduction

Preclinical imaging instrumentation has substantially improved over the past decade (1). Currently, many multimodality scanners that have been customized for rodent imaging are available. The molecular imaging modality that is best for detecting and quantifying small amounts of exogenously administered biomarker material is Positron Emission Tomography (PET). PET has the best combination of sensitivity and ability to image deep within the tissue of any existing preclinical/clinical imaging modality and has also good spatial resolution. Hybrid PET and MRI (Magnetic Resonance Imaging) systems provide simultaneously both anatomical (with excellent soft tissue contrast capability) and molecular imaging information (2). There are several research and commercially available preclinical PET/MR scanners. PET instrumentation typically relies on multiple detector modules optimized for 511 keV annihilation photons detection arranged in an annular or multi-panel geometry. Other geometries have also been described in the literature (3–9). These modules in most cases are built on the basis of a scintillation crystal block and a photosensor array. For simultaneous PET/MR imaging, solid-state photosensors are employed. Today Silicon Photomultipliers (SiPM) are the most common type of photosensor technology for this task. Most PET scanners make use of scintillators based on a pixelated design in which the raw scintillator crystal is cut into small elements (pixels) to produce arrays of optically isolated pixels in order to spatially localize the scintillation event in the crystal block. The basic 511 keV photon interaction localization is done in the 2D coordinates of the pixel array. Photon Depth of Interaction (DOI) can be used to estimate the photon interaction position in the third dimension. DOI is achieved using multiple layers of crystal pixel arrays either staggered or made of different scintillator material [phoswich type (10–13)]. More involved and complicated designs make use of double-sided readout with additional photo-sensors at the gamma entry faces to accurately deduce the DOI (14).

A more attractive and elegant alternative to crystal pixelation is the use of flat monolithic, non-pixelated scintillator crystals (9, 15). This technology has been indeed used not only in preclinical systems but also in dedicated human scanners for brain (16–18) or breast imaging (19). The 3D photon impact coordinates are extracted with high precision from the shape of the scintillation light distribution measured at the photo-detector surface. As in the case of the pixelated variant, double-sided readout schemes were also proposed to obtain better DOI resolution but also to get better TOF definition (20).

**Figure 1:**
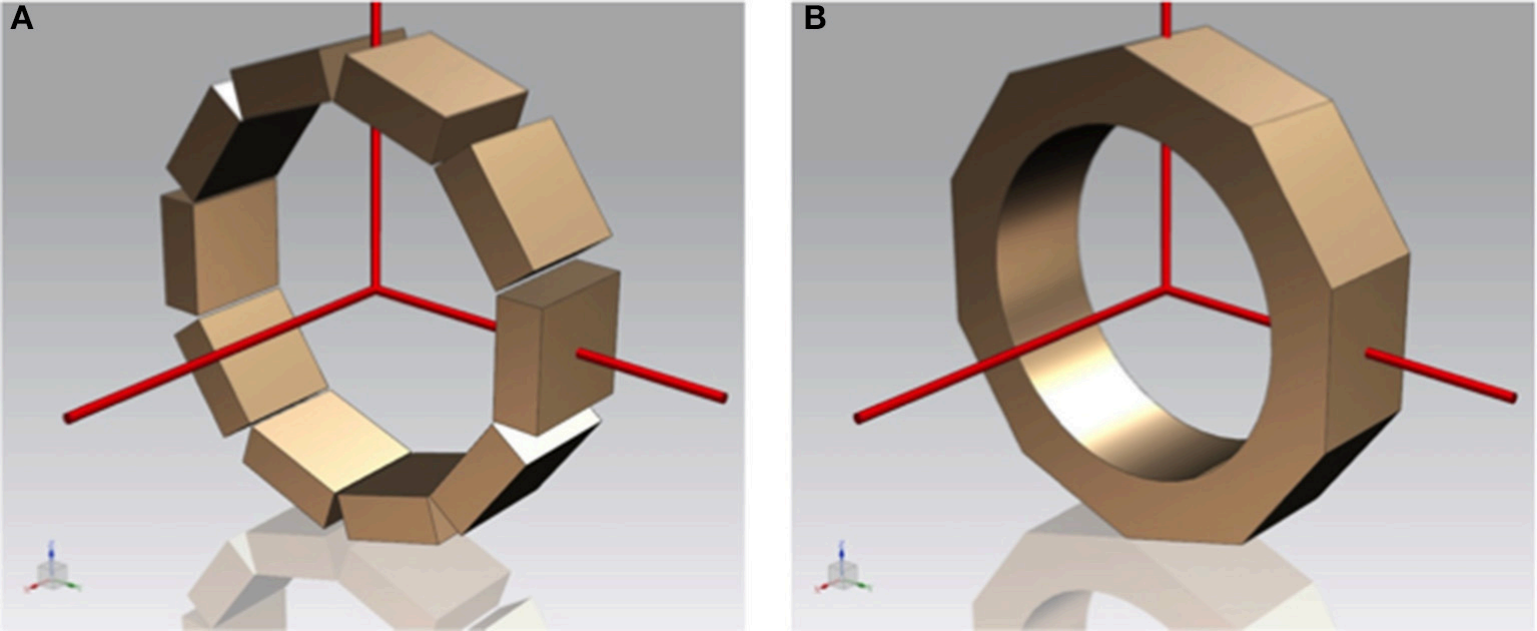
Sketches of a PET scanner based on individual modules **(A)** and built as a single monolithic annulus-like crystal **(B)**.

The use of separate detector modules, independently of the technology they are based on, results in physical gaps between the modules (see Figure 1 left). These gaps can be significantly reduced given the compact high granularity photosensor technology such as SiPMs, but cannot be entirely eliminated. In addition, there is a dependence of the detector spatial and energy resolution performance on the photon conversion position for both monolithic modules and crystal arrays. For monolithic scintillator blocks this is due to the scintillation light truncation which is more prominent at the crystal edges.

We are proposing to build a PET insert scanner, compatible with high-field MR systems, for small animals based on a single annulus-like scintillator, therefore reducing the number of scintillator blocks to one and thus, avoiding the multiple gaps, as shown in Figure 1B. By implementing one single scintillator volume, we eliminate the edge effects in the transaxial plane, but also gaps in the angular coverage. Therefore, this design increases the system's sensitivity and uniformity of response. While this design is novel, there is already some prior art confirming the timely importance of this subject (21, 22).

In the following we describe studies carried out with an existing system in which we simulate minimization of the edge effects to assess the performance improvement. Next we describe the proposed design geometry and results of both nuclear and optical simulations proving the advantages of building such a novel uniform-response system.

## Materials and methods

### Experimental proof of concept using existing multi-module system

First, in order to study dependence of energy and spatial resolution on the impact position in the scintillation volume, we carried out experiments with monolithic LYSO scintillation blocks with trapezoidal shape and dimensions of about 48 ×48mm at the front and 50 × 50mm at the back, and 10mm thickness. A small size ^22^Na source was moved across one of the axes of the detector. The source was 0.25mm in diameter and was directly placed in front of the crystal and moved with steps of 0.5mm. No mechanical collimation using high atomic number masks, such as made from tungsten or lead, was applied. To operate in coincidence mode, an identical opposite detector was placed at a distance of 11.5 cm. To better evaluate the performance of these blocks, a software beam collimation of 2.1° from the normal was applied in this system. As it will be explained in the results section, a deterioration of the detector block performance is observed at the edges.

To show the benefits of an edgeless PET scanner, we acquired experimental data of a mini Derenzo phantom (rods starting at 0.75mm) and compared the resolution in the resulting reconstructed images with that obtained using the same data but excluding coincident events with one or both annihilation photons detected near a crystal edge. The coincidence data set was acquired using a prototype of a PET insert from Bruker (23). The system is composed from 3 rings of 8 of those described trapezoidal monolithic LYSO scintillations each (simulating 2 axial and 8 transaxial gaps, respectively). All the crystal faces, except the one in contact with the photosensor were black painted in order to preserve as much as possible the scintillation light distribution within the crystal. The trapezoidal shape helps reducing gaps in between blocks, and at the same time improves the event detectability by reducing edge effects. SiPM arrays of 16 × 16 SiPM with active area of 3 × 3mm each and 3.26mm pitch were used. The 3 rings system has roughly 150mm axial and 80mm transaxial field of views, respectively. The insert was installed at the University of Leuven, Belgium.

**Figure 2:**
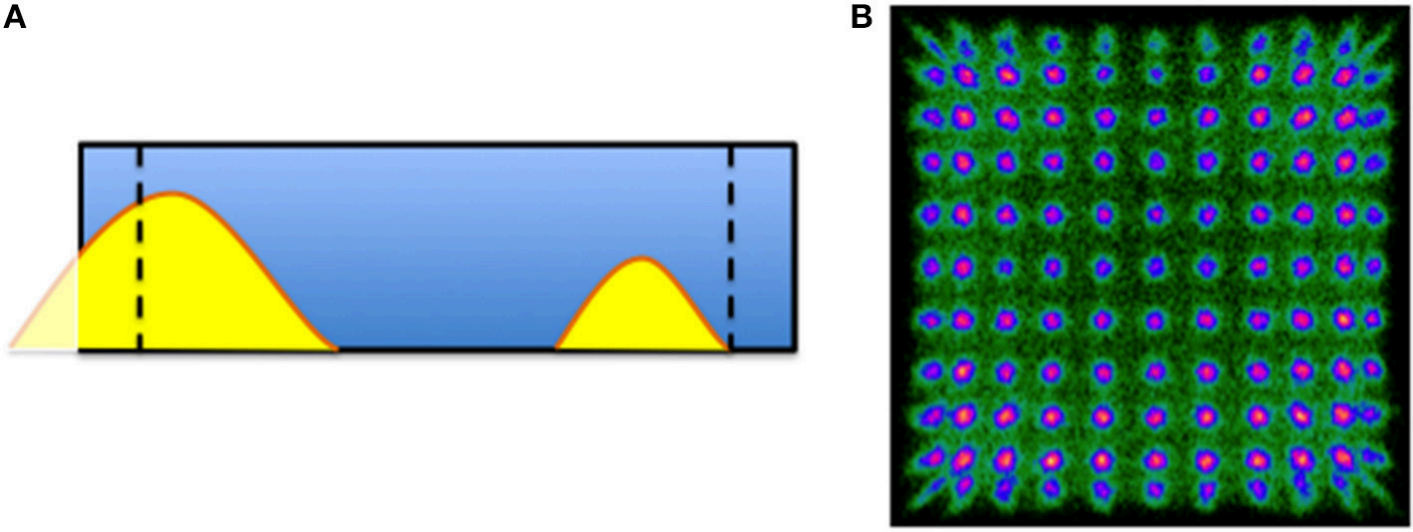
**(A)** Sketch of the crystal with two scintillation light distributions, the dashed lines represent the 60% of the crystal volume. The flood map in **(B)** shows the 11 × 11 collimated positron-emitter sources.

PET images obtained with the above PET insert were reconstructed using lines of response (LORs) that included impacts in the entire volume of the detector blocks (*Original*) and also with LORs involving only the inner 60% of each block (*Filtered*), see Figures 2A,B. The flood map shown on Figure 2B illustrates the resulting detector image after measuring with an 11 × 11 array of ^22^Na sources (pitch of 4.6mm) with sources as close as 2mm to the crystal edge. The yellow dashed line roughly depicts the 60% region considered in the filtered reconstruction, approximating the proposed continuous-tube detector behavior by removing impacts near the crystal edges. We reconstructed the collected data using Maximum Likelihood Expectation-Maximization (MLEM) algorithm with multiple graphics processing processors. We used 35 iterations and regular voxel and virtual pixel sizes of 0.25 and 1.5mm, respectively.

### Design approach, simulations, and initial reconstruction

**Figure 3:**
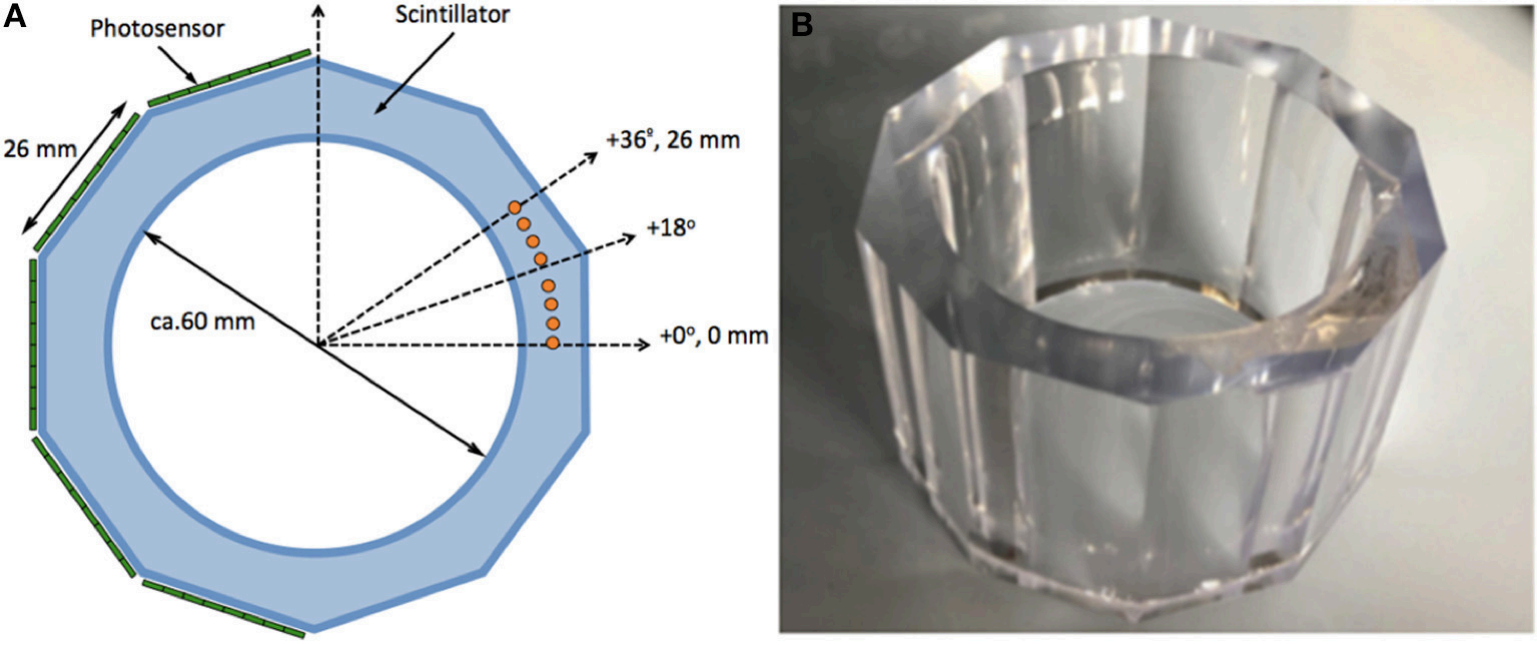
**(A)** Sketch of the design with 10-face scintillation tube. **(B)** Photograph of the manufactured monolithic LYSO tube.

We have designed a single LYSO scintillator crystal with cylindrical-like shape but with ten facetted external exit faces. Inner diameter was selected as about 60mm and the largest outer diameter of about 80mm. These dimensions are a compromise between expected system performance and compatibility with existing RF coils for research studies with rodents. Truncated exit faces allow for an easy photodetector coupling with current SiPM technology (see Figure 3A). Alternative implementations could include also circular exit faces and SiPMs mounted on a flexible printed circuit board. The axial length is planned to be about 80mm, allowing simultaneous imaging of an entire mouse. We have consulted several LYSO scintillation manufacturers and these dimensions are feasible since standard ingot sizes are about 85mm diameter and 120mm length. Figure 3B shows a picture of an already manufactured LYSO tube by Proteus (Ohio, USA). Price-wise, manufacturing an LYSO tube like this might be about 20% cheaper than a similar geometry covered with crystal arrays of 1mm size and 10mm height, but about twice more expensive than with monolithic blocks of 1 inch size.

Regarding photosensor and readout implementation, SiPMs with 3 × 3mm active area, and very small gaps (100–200 μm), are planned. Different vendors provide this technology as standard or customized products. Different readout technologies might be utilized namely digitizing every photosensor SiPM element using for instance Application Specific Integrated Circuits (ASICs) or an analog integrated approach providing information for each row and column signals of the SiPM array (9, 17). A significant additional requirement resides in the MR compatibility. We have considered the inner and outer diameters to fit the standard RF coils, as well as some gradient coils. Moreover, the proposed design would include RF shielding based on carbon fiber structures, as already successfully demonstrated by our team before (21).

We have implemented the described geometry in GATE (v7.2) simulation software (24, 25). We set a time resolution of 1 ns and a coincidence window of 2 ns. An energy window of 15% at the 511 keV photopeak was selected. Only double coincidences were allowed. Multiple crystal interactions (scatter) are included in this data with the average derived energies and positions. We examined only the nuclear interactions. That means that the scintillation light propagation effects were not included in this simulation, but only the interactions within the crystal volume. In order to improve simulations, we applied a common impact position blurring of 1mm in the planar impact and 2mm in the depth of interaction, based on existing data from similar scanner designs (9). Data was also binned in 3mm, as we expect to use 3 × 3mm SiPM array in the system.

Several phantoms have been simulated such as a small spherical source (0.25mm diameter), as well as a line source covering the full axial length (26mm), both placed at the center of the FOV. We also implemented the mouse Noise Equivalent Count Rate (NECR) phantom as suggested by the NEMA protocol NU 04-2008 for two axial lengths 26 and 52mm, and for the two designs shown in Figure 1 namely toroid and multiple crystals, respectively. An expected parallelized deadtime for the electronic of about 700 ns was added. Generated simulation data has been first reconstructed using list-mode Ordered Subsets Expectation Maximization (OSEM), in order to prove the feasibility from the reconstruction point of view. Two point sources, separated by 15mm, have been simulated and reconstructed (4 iterations and 10 subiterations). To further prove such design concept, we have simulated an annulus (3 × 10^6^ events) to estimate the detector pair sensitivities and create a system normalization matrix, and a Derenzo-like phantom with rods of 2.5, 2, 1.5, 1.25, and 1mm in diameter. The annulus had inner and outer diameters of 49 and 50mm, respectively, placed in the center of the FOV, see Figure **6C**. The simulated Derenzo-like phantom is based on polymethyl methacrylate (PMMA) material and labeled with ^18^F. It was reconstructed using list-mode MLEM with 40 iterations.

### Light propagation and characterization

We have studied through simulations how the scintillation light spreads out for the suggested crystal design. Using GATE, we have analyzed light distributions for different impact positions. At each impact position 16,000 optical photons (2.96 eV) were generated and emitted in random directions. We have considered the inner circular scintillator face as well as the lateral faces painted black (95% absorption and 5% Lambertian diffusion). Coupling between the scintillation tube and the photosensors was carried out using optical grease (n_grease_ = 1.4, n_LYSO_ = 1.8). We have studied the projected light distributions following channel-reduction approaches developed before, instead of reading each photosensor individually (9, 17, 26). The axial projection would be used both to locate the impact coordinates and to provide DOI information (9, 26). We have sampled the scintillation light distribution with 3mm pixels, as we plan to use 3 × 3mm SiPMs. Notice that the axial axis in these simulations was reduced to only 26mm, in order to test that the design would be also valid for small axial crystal lengths.

First, we placed sources at different angular positions and at a DOI of 4.5mm from the inner circular surface. The sources were at angles from 0° to 36° (corresponding to the centers of two adjacent facets), as sketched in Figure 3A with solid orange circles. The virtual line separating two photo-detector sections is at a multiple angle of ± 18°. Therefore, impact positions at for instance 16° or 20° belong to interactions to the left and right of this virtual line, respectively. We studied the accuracy in the determination of the centroid, but also the linearity in terms of the relation between the known and measured positions. A second set of simulations was performed to show the design capabilities to extract DOI information.

## Results

### Experimental proof of concept using existing multi-module system

**Figure 4:**
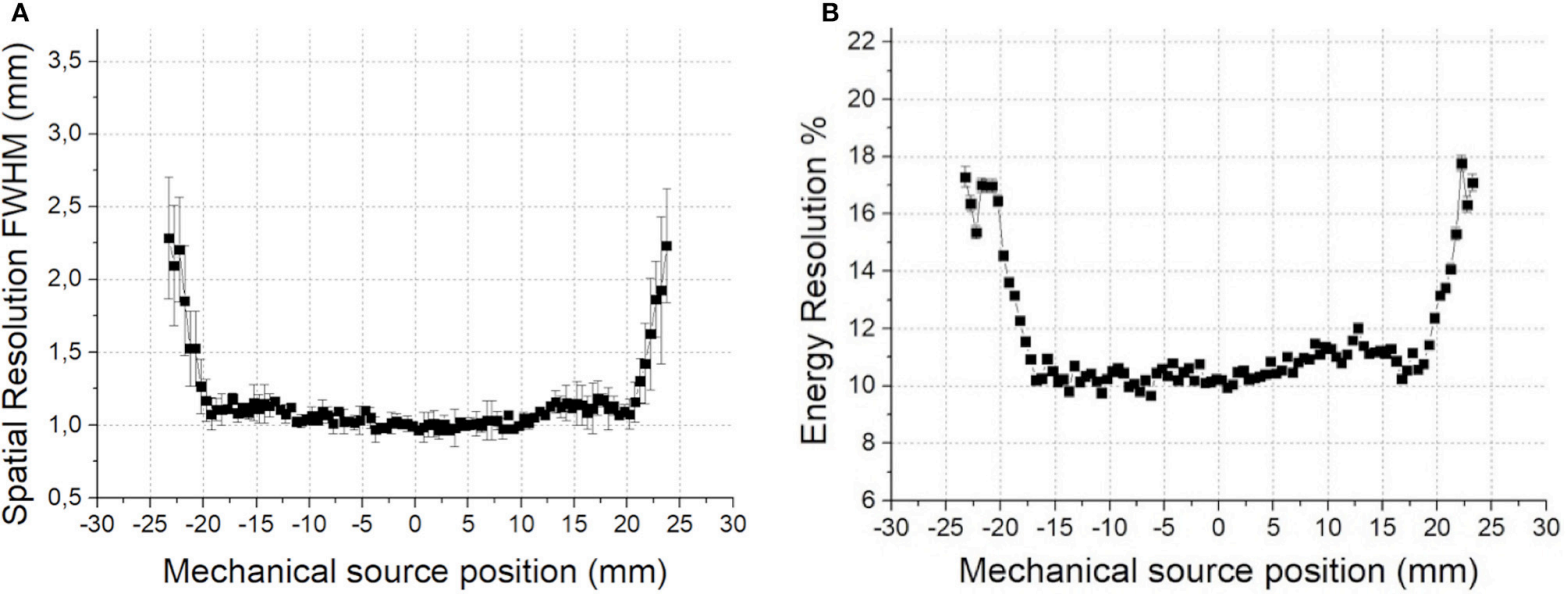
**(A)** Measured spatial resolution in a 10mm thick monolithic block as a function of the impact position. **(B)** Energy resolution dependence.

The results obtained when analyzing the performance of the individual detector blocks are shown in Figure 4A. It depicts the measured spatial resolution (including source size) as the full width at half of the maximum (FWHM) of the source profiles, as a function of the ^22^Na source impact position. Overall, crystals have shown good performance in terms of spatial, energy and depth of interaction (DOI) resolutions (9). However, due to the light truncation at the edges, these parameters exhibit some deterioration there. Measured spatial resolution remains almost constant for most of the crystal area and is close to 1mm. However, a degradation to 2mm is observed at the very edges. Also, energy resolution exhibits a similar behavior worsening from 10% at the crystal center to 14% at 20mm off-center, as shown in Figure 4B.

**Figure 5:**
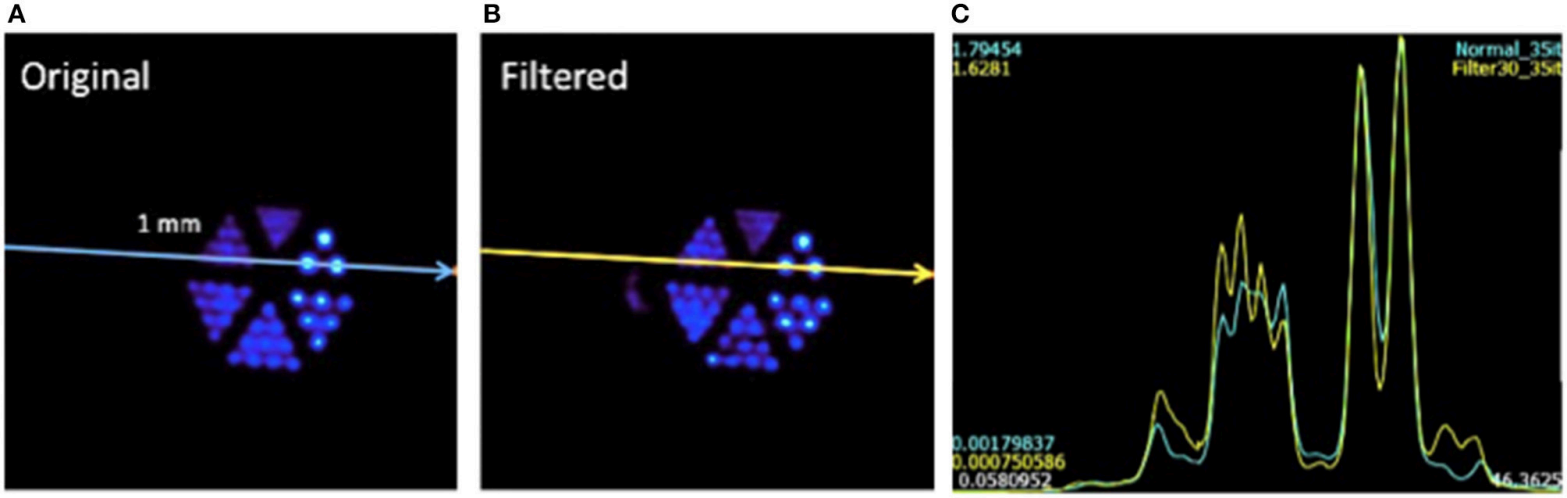
**(A)** Reconstructed Derenzo-like phantom images using all crystal impacts. **(B)** The same reconstruction considering data only in the 30 × 30mm central region. **(C)** Profiles for both cases (yellow line is for the filtered case).

Regarding the PET images obtained with the Bruker PET insert, profiles through the hot rods of the measured mini Derenzo were obtained and fitted with Gaussian curves. For rods of 1mm in diameter the fitted Gaussian width was 1.45mm FWHM on average for the original data set and 1.19mm for the filtered data set, roughly a 25% increase in resolution. Figure 5 shows the reconstructed images obtained under the original and filtered conditions, Figures 5A,B, respectively. Profiles across the 1mm rods are shown in Figure 5C.

These results suggest an improvement in the achievable image resolution of 25%. Therefore, based on current distinguishable rods of 0.75mm in a micro Derenzo phantom, image resolution nearing 0.6mm would be possible. Notice this would be feasible when using tracers with low positron energies such as ^18^F (634 keV), which average positron ranges is 0.5–0.6mm.

### Design approach, simulations, and initial reconstruction

Using the proposed geometry, a system sensitivity of 5.3 and 2.9%, was simulated for the small spherical 0.25mm source and for the line source covering the full axial length, respectively (axial length 26mm in both cases). In the case of a PET design based on multiple detectors, as the one shown in Figure 1A, the calculated sensitivities decrease to 3.7 and 2.0%, respectively (about 30% reduction). The results obtained for the NECR curves exhibited a maximum peak at about 1 mCi of 116 kcps and 509 kcps, for the toroid approach for 26 and 52mm axial length, respectively. For the multiple crystals, we obtained 87 and 353 kcps.

**Figure 6:**
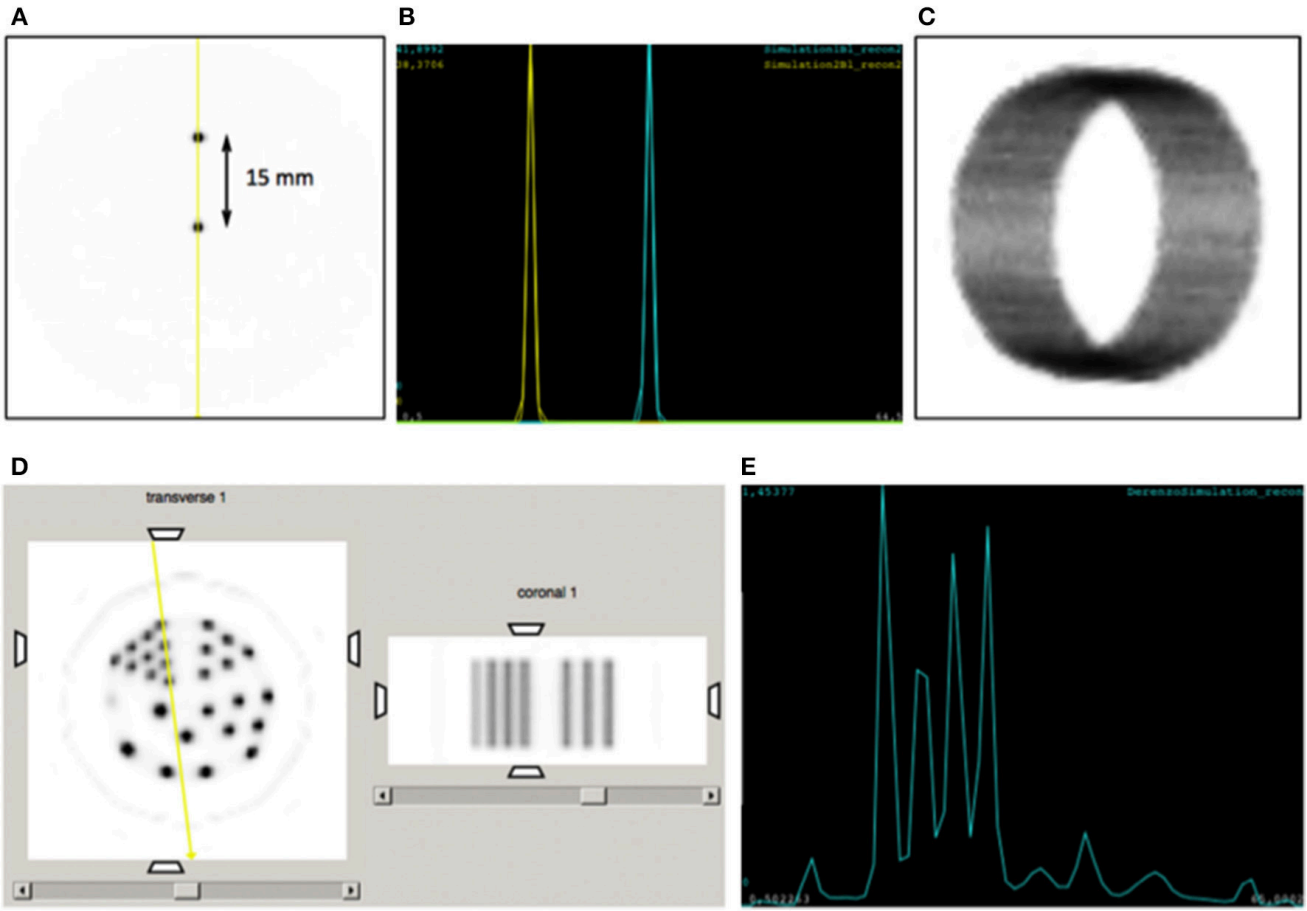
**(A)** Image reconstruction of two point sources, 15mm separated. **(B)** Lines profiles across the two sources. **(C)** 3D reconstruction of the normalization annulus. **(D)** Reconstruction of the Derenzo-like phantom (transverse and coronal views). Notice that one of the hot rods was filled with 10% of the concentration in the other rods. **(E)** Line projections across the smallest rods.

Figures 6A,B show the simulation results of reconstructed point sources (separated by 15mm) and the line profiles through the sources, respectively. And, Figures 6D,E shows the reconstruction of the Derenzo-like phantom and the line projections across the smallest rods, as depicted all rods were well resolved. These results demonstrate the performance application of both OSEM and MLEM reconstruction algorithms. In addition, since both the sources and the smallest rods are clearly resolved, high resolution values can be expected.

We have therefore demonstrated the feasibility of reconstructing images obtained through GATE simulations for a system based on a single toroid crystal. In those reconstructions only the nuclear hits contributions were considered.

### Light propagation and characterization

**Figure 7:**
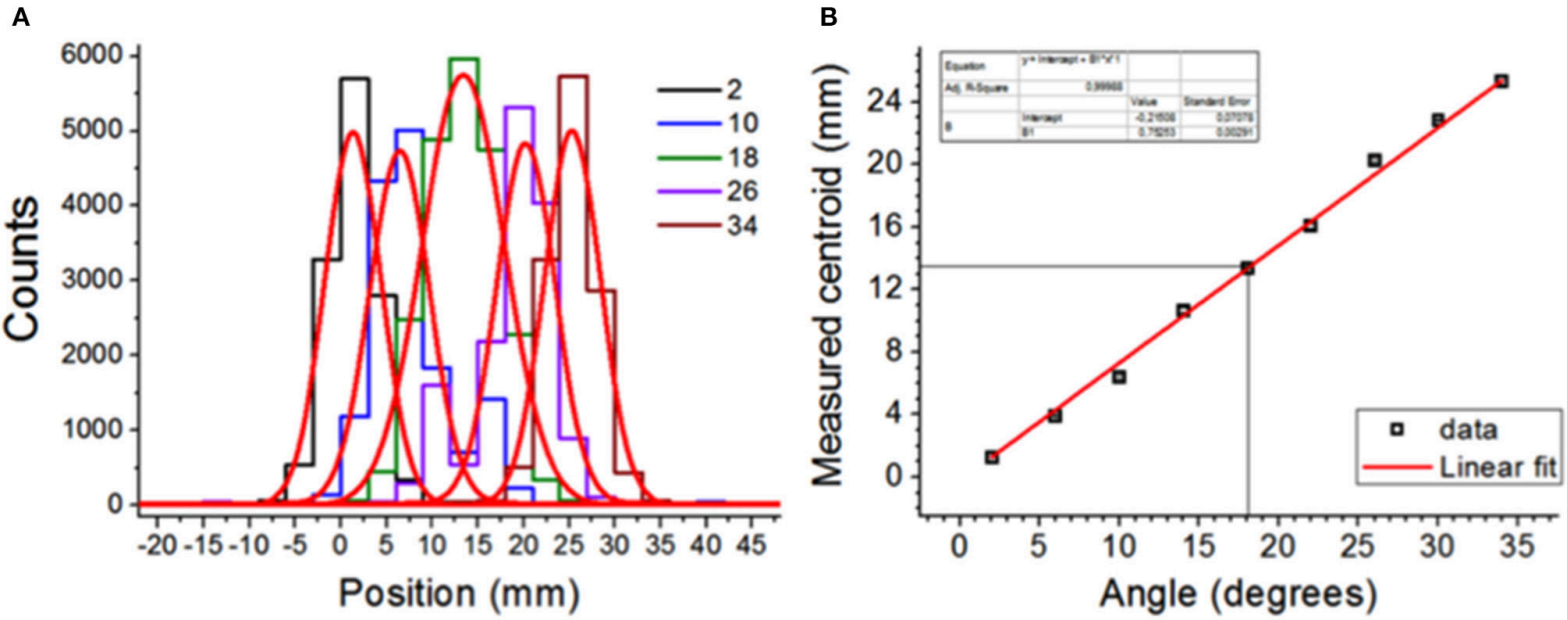
**(A)** Light distributions using 3mm bins for different impact positions (in angles). The red lines are Gaussian fits. **(B)** Measured centroid as a function of known position (in angle) showing a good linearity without edge effects.

Figure 7A shows the determined light distributions for the simulated sources at different angular positions and at a fixed DOI of 4.5mm, together with their corresponding Gaussian fits. Narrower distributions are observed at 0 and 26mm, since these are the centers of the facetted faces. However, a wider distribution is observed at 13mm, the position between two neighboring facetted faces. In Figure 7B, we have plotted the calculated centroid position as function of the known simulated position (in angle). It can be seen that there is no compression effect. This shows we can unambiguously retrieve the impact position in this direction.

**Figure 8:**
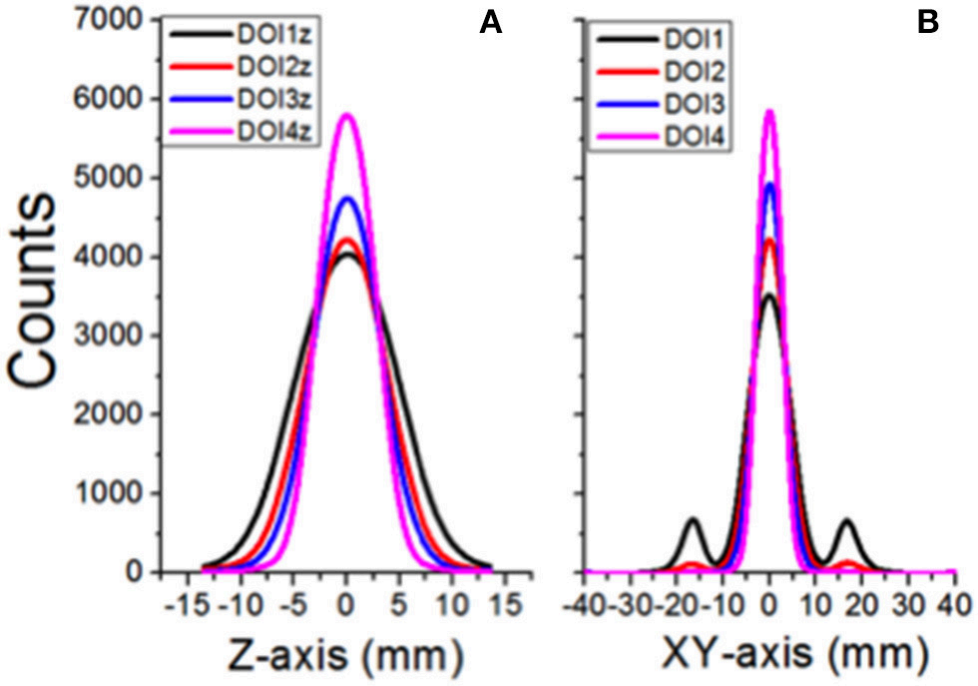
Light distributions for different DOI impact positions in the crystal, projected onto the axial **(A)** and transaxial planes **(B)**, respectively.

The results for the simulation showing the system performance extracting the DOI information are depicted in Figure 8. Light distributions at different DOI (1, 3, 5, and 8mm measured from the entrance scintillator surface) for both the axial z-axis (a) and the transaxial coordinates (b) are shown.

In summary, and in contrast to the plots observed in Figure 4, we expect a homogeneous Y (transaxial direction) resolution of 1mm for all impact points.

## Discussion and conclusions

The purpose of this initial work was to perform a feasibility study of a novel design for a small animal PET imager, based on a single monolithic scintillation LYSO tube. Simulations and experimental reconstruction data confirm the feasibility of the concept.

Initially proposed dimensions are: ~80mm outer diameter, ~60mm inner diameter and ~80mm axial length. Benefiting from its intrinsically MR compatible components, this PET scanner could be employed as an insert in existing small animal MR scanners to form a high performance PET/MR scanner. Sixty millimeter inner diameter is selected to operate with the existing radio-frequency coils.

In addition to simulations, we have also demonstrated experimentally the benefits of the continuous scintillator design in simulated reconstructed images by suppressing photon conversion points near the crystal edges in a real case based on a ring of planar monolithic blocks. The measured spatial resolution for the Bruker PET insert using all the events, including the ones at the edges is 0.75mm. Based on current results, the resolution of an edgeless detector system is estimated to be 0.6mm for the whole FOV using an accurate photon impact DOI determination. This will allow achieving a homogeneous spatial resolution for a whole mouse volume that is only limited by the positron range (average distance the positron travels before annihilation; for ^18^F, it is about 0.6mm). Moreover, since our plan is to use this PET design as an insert for high-field MRI, some further improvement due to the positron confinement could be expected (27).

Several scintillator manufacturers have assured us that it is possible to fabricate such single-crystal tubes, as it can be seen in Figure 3B. We expect a final cost of the crystal tube similar to that achieved when independent blocks are purchased. We are aware of two other groups working on a similar approach, and this additionally confirms the viability of our approach (21, 22). We are therefore confident that the system can deliver high precision 3D point of interaction determination with DOI accurately extracted from the axial projection. We do not observe deterioration in the XY determination due to scattered light.

## Author contributions

AG, SB, and SM contributed with concepts and the design of the study. CC and AO prepared the existing data for reconstruction without edges. AG-M, GC, FS, and JB performed the statistical analysis of light distributions in the new crystal geometry. GC, AR, and JN simulated and reconstructed the data with the proposed new geometry. AG wrote the first draft of the manuscript. SM reviewed and edited the overall manuscript. All authors contributed to manuscript revision, read, and approved the submitted version.

### Conflict of interest statement

The authors declare that the research was conducted in the absence of any commercial or financial relationships that could be construed as a potential conflict of interest.
